# The Jumonji-domain histone demethylase inhibitor JIB-04 deregulates oncogenic programs and increases DNA damage in Ewing Sarcoma, resulting in impaired cell proliferation and survival, and reduced tumor growth

**DOI:** 10.18632/oncotarget.26011

**Published:** 2018-09-04

**Authors:** Janet K. Parrish, Tyler S. McCann, Marybeth Sechler, Lays M. Sobral, Wenhua Ren, Kenneth L. Jones, Aik Choon Tan, Paul Jedlicka

**Affiliations:** ^1^ Department of Pathology, Anschutz Medical Campus, Aurora, CO, USA; ^2^ Cancer Biology Graduate Training Program, Anschutz Medical Campus, Aurora, CO, USA; ^3^ Department of Pediatrics, Anschutz Medical Campus, Aurora, CO, USA; ^4^ Department of Medicine, Anschutz Medical Campus, Aurora, CO, USA; ^5^ University of Colorado Denver, Anschutz Medical Campus, Aurora, CO, USA

**Keywords:** Ewing Sarcoma, epigenetics, Jumonji, demethylase, inhibitor

## Abstract

Ewing Sarcoma is an aggressive malignant neoplasm affecting children and young adults. Ewing Sarcoma is driven by transcription factor fusion oncoproteins, most commonly EWS/Fli1. While some patients can be cured with high-dose, multi-agent, chemotherapy, those that cannot currently have few options. Targeting of the driver oncofusion remains a logical therapeutic approach, but has proven difficult. Recent work has pointed to epigenetic mechanisms as key players, and potential new therapeutic targets, in Ewing Sarcoma. In this study we examined the activity of the pan-JHDM pharmacologic inhibitor JIB-04 in this disease. We show that JIB-04 potently inhibits the growth and viability of Ewing Sarcoma cells, and also impairs tumor xenograft growth. Effects on histone methylation at growth-inhibitory doses vary among cell lines, with most cell lines exhibiting increased total H3K27me3 levels, and some increased H3K4me3 and H3K9me3. JIB-04 treatment widely alters expression of oncogenic and tumor suppressive pathways, including downregulation of known oncogenic members of the Homeobox B and D clusters. JIB-04 also disrupts the EWS/Fli1 expression signature, including downregulation of pro-proliferative pathways normally under positive oncofusion control. Interestingly, these changes are accompanied by increased levels of the EWS/Fli1 oncofusion, suggesting that the drug could be uncoupling EWS/Fli1 from its oncogenic program. All Ewing Sarcoma cell lines examined also manifest increased DNA damage upon JIB-04 treatment. Together, the findings suggest that JIB-04 acts via multiple mechanisms to compromise Ewing Sarcoma cell growth and viability.

## INTRODUCTION

Ewing Sarcoma is a biologically and clinically aggressive cancer of bone and soft tissue predominantly affecting the pediatric age group [1]. The current mainstay of Ewing Sarcoma treatment is high-dose, multi-agent chemotherapy, which can cure roughly two thirds of patients, mainly those presenting with non-metastatic disease [1]. However, those who fail chemotherapy, including the majority of patients presenting with metastatic disease or/and recurrence, face very poor outcomes, and have few treatment options [1].

The pathogenesis of Ewing Sarcoma is driven by EWS/Ets fusion oncoproteins, most commonly EWS/Fli1, which arise as a consequence of recurrent chromosomal translocations [1–3]. EWS/Ets oncofusions are aberrant transcriptional and post-transcriptional regulators, which cause widespread dysregulation of gene expression, ultimately leading to a malignant neoplastic phenotype [2, 3]. As disease drivers, EWS/Ets oncofusions represent logical therapeutic targets in Ewing Sarcoma, but, to date, such targeting has proven difficult [4, 5]. A number of laboratories, including ours, have sought to identify alternative targetable molecules and pathways in Ewing Sarcoma [4, 5]. Many recent studies in the field of pediatric cancer have highlighted the importance of epigenetic mechanisms in disease initiation and progression [6], while epigenetic regulators themselves have attracted substantial interest as a new category of tractable therapeutic targets in cancers of all types [7]. Epigenetic mechanisms have recently emerged as very important players in Ewing Sarcoma pathogenesis [8–12], and a number of studies have identified pathogenic roles for specific chromatin modifiers in the disease [13–15]. Our own studies of microRNA-mediated mechanisms of EWS/Fli1-driven oncogenesis led us to the discovery of a tumor promotional role for a member of the Jumonji-domain histone demethylase family [16–18].

Jumonji-domain histone demethylases (JHDMs) comprise a large family of proteins (approximately twenty in humans) that share a homologous Jumonji domain, and an enzymatic demethylation mechanism involving Fe^2+^ and α-ketoglutarate [19, 20]. Individual members of this family have unique and overlapping specificities for methylated histone residue substrates [19, 20]. As true of other epigenetic regulators, therapeutic targeting of JHDMs has generated substantial recent interest [21, 22]. Notably, a compound has recently been identified in a chemical library screen that possesses potent activity against JHDMs and manifests anti-tumor activity *in vivo* [23]. In the present study, we undertook evaluation of this compound (JIB-04) in Ewing Sarcoma.

## RESULTS

### JIB-04 potently inhibits Ewing Sarcoma cell and colony growth

**Figure 1:**
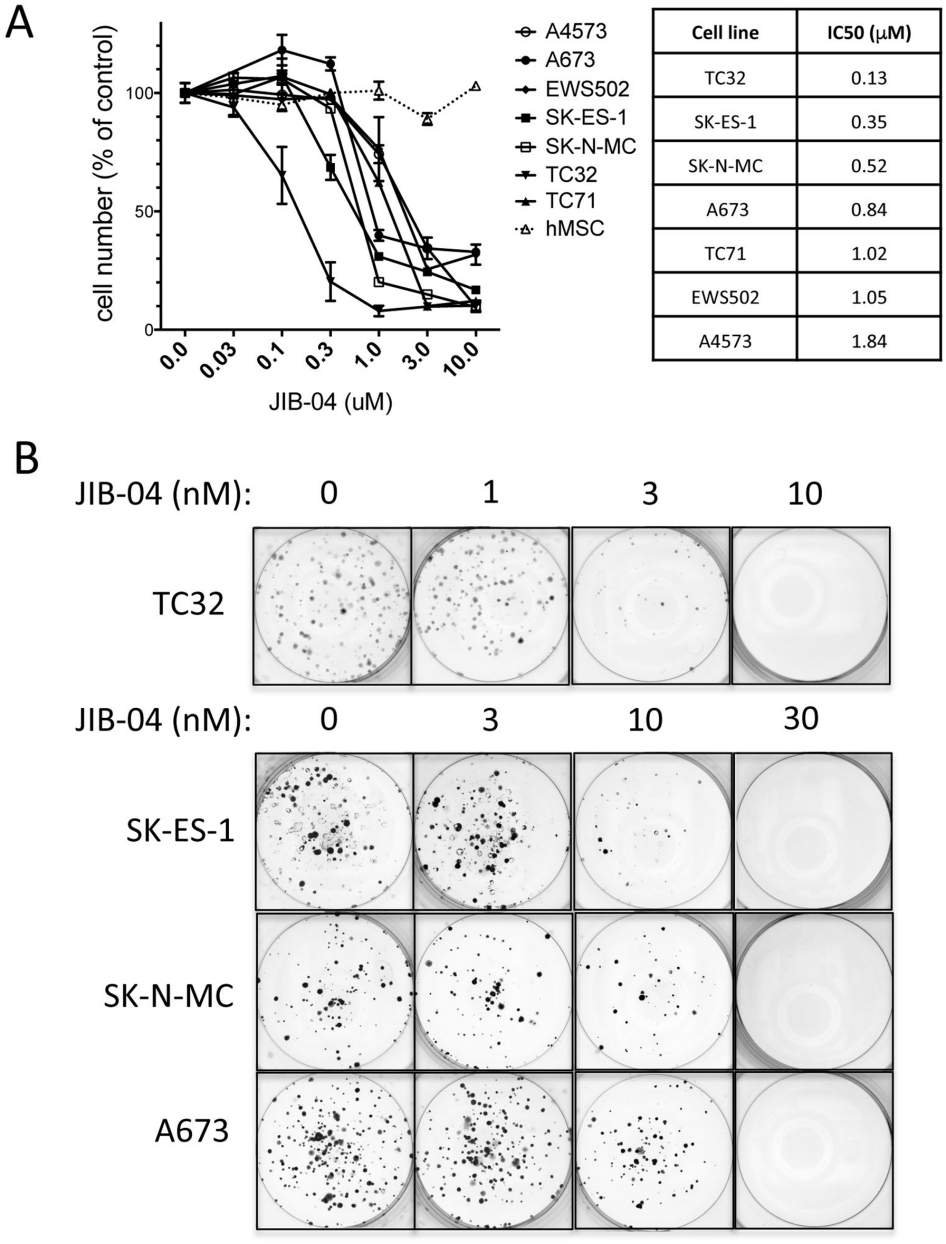
Growth inhibitory activity of JIB-04 in Ewing Sarcoma **(A)** One day following plating, the indicated cells (7 different Ewing Sarcoma cell lines, and human mesenchymal stem cells (hMSC)) were treated for 48 hours with the indicated concentrations of JIB-04. Cell numbers at the end of the experiment were quantified using an MTT assay, and were normalized to vehicle-treated cells. Results represent the mean and standard error of the mean (SEM) of at least 2 independent experiments, each performed in replicate. IC50 values for growth/survival inhibition of Ewing Sarcoma cells by JIB-04, calculated from the data in panel A, are shown on right. **(B)** Beginning one day following plating (500 cells per well), cells were treated with the indicated concentrations of JIB-04 (or vehicle control) every 2 days. Colonies were visualized by crystal violet staining approximately 2 weeks later. Representative images of triplicate platings are shown.

In order to determine whether the Jumonji-domain histone demethylase (JHDM) inhibitor JIB-04 affects the growth of Ewing Sarcoma cells, we examined its activity against a panel of patient-derived Ewing Sarcoma cell lines in an *in vitro* drug sensitivity assay. Cells were plated at a concentration to ensure logarithmic growth during drug exposure, and, beginning 16 hours post-plating, were treated with drug (or vehicle control) for 48 hours, at which point viable cell numbers were measured using an MTT assay [24]. This analysis revealed growth inhibitory activity of JIB-04 against all cell lines tested, with IC50 values ranging from 0.13 μM (TC32 cells) to 1.84 μM (A4573 cells) (Figure 1A). In contrast, JIB-04 did not inhibit the growth of normal primary human mesenchymal stem cells (hMSC), the putative cell of Ewing Sarcoma origin, in the same assay (Figure 1A).

We next asked whether growth under low-density culture conditions would result in even greater drug sensitivity. To this end, we examined the effects of JIB-04 in a low-density culture clonogenic assay. We treated TC32, SK-ES-1, SK-N-MC and A673 cells with vehicle or drug beginning one day following plating at 500 cells per well, and colonies were visualized approximately 2 weeks later [24]. Under clonogenic growth conditions, JIB-04 inhibited Ewing Sarcoma colony growth in the low nanomolar range (Figure 1B). Thus, JIB-04 manifests growth inhibitory activity against Ewing Sarcoma cells, but not hMSCs, under high-density culture conditions, and potently inhibits Ewing Sarcoma clonogenic growth at low-density culture conditions.

### Changes in histone methylation in response to JIB-04

**Figure 2:**
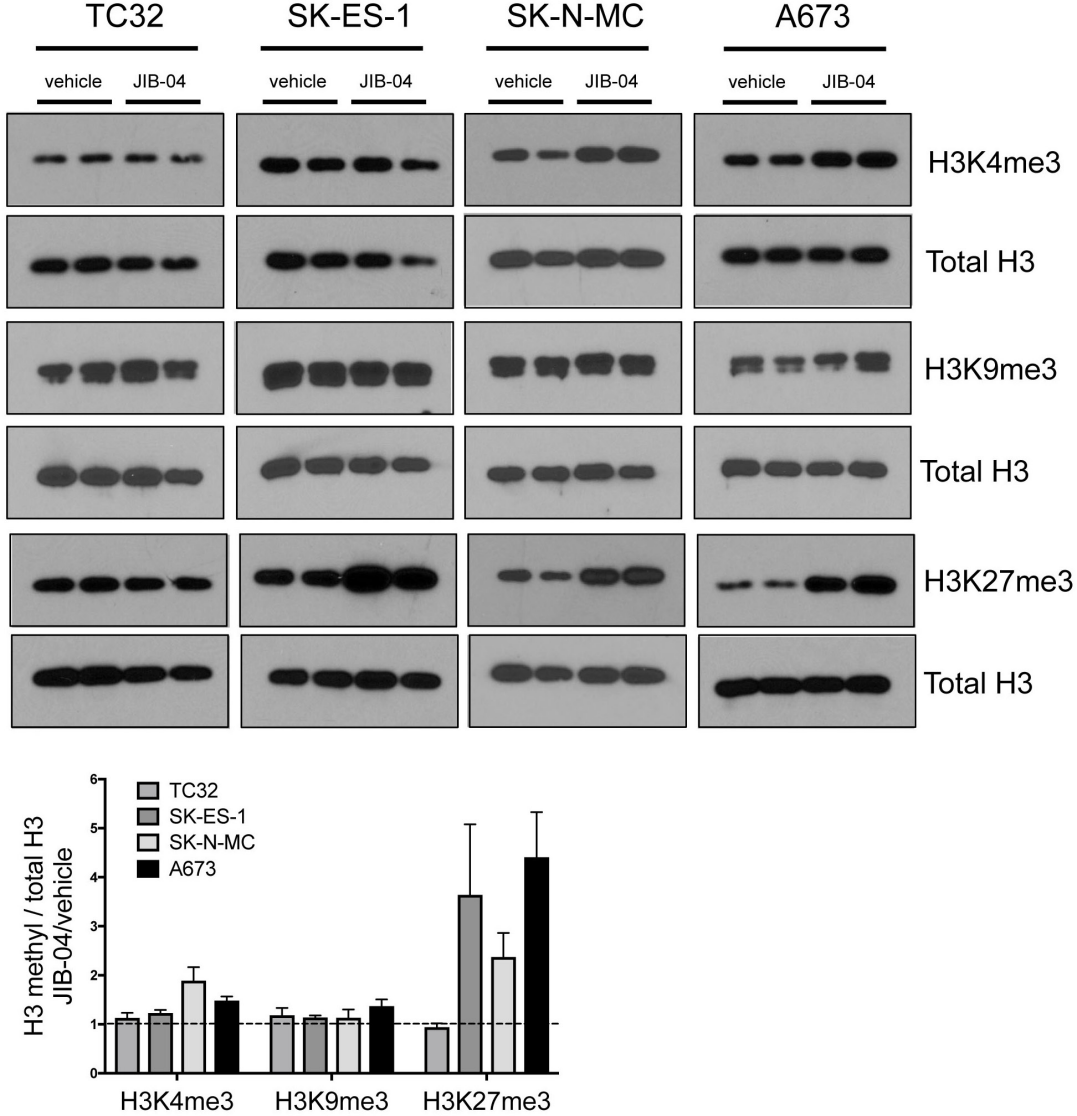
Effects of JIB-04 on global histone methylation in Ewing Sarcoma cells Levels of the indicated histone methyl marks, in vehicle and JIB-04-treated cells (TC32: 0.3 μM; SK-ES-1 and SK-N-MC: 1 μM; A673: 2 μM), as determined by immunoblotting of acid-extracted histones collected at 36 hours following treatment. Representative blots at the top. Shown below is densitometric quantification of histone methyl marks levels, normalized to total histone H3 levels, and plotted relative to vehicle-treated cells (mean and SEM of 2-3 independent experiments, each done in duplicate).

Prior characterization of JIB-04 indicates that it has the potential to inhibit multiple JHDMs [23], resulting in its classification as a pan-Jumonji histone demethylase inhibitor. Human cells contain approximately 20 different JHDMs, with distinct and overlapping specificities for different histone methyl marks [20, 21]. The majority of JHDMs show activity against one or more methyl marks on histone H3 residues K4, K9 and K27 [25], all of which have been implicated in regulation of gene expression (K4 methylation at promoters being permissive/ promotional to gene expression, and K9 and K27 methylation at promoters being inhibitory; [26]). To begin to get insight into potential mechanisms of action of JIB-04 in Ewing Sarcoma cells, we examined global levels of methylation at these residues (Figure 2). We focused on tri-methyl marks, which have been most extensively studied with respect to gene regulation and other cellular functions. Analysis was performed at drug doses slightly above the IC50 for each cell line, and at a time (36 hours) prior to the end point of the high-density growth assay (48 hours of drug treatment), in order to reflect changes occurring under growth inhibitory conditions. Under these conditions, JIB-04 treatment resulted in increased global H3K4 trimethylation in SK-N-MC cells (∼2-fold increase) and A673 cells (∼1.5-fold increase). Global H3K9 trimethylation showed a slight increase in A673 cells, but not the other cell lines. Global H3K27 trimethylation showed the most dramatic changes, increasing 2-4 fold in SK-ES-1, SK-N-MC and A673 cells. TC32 cells, growth-inhibited by JIB-04 at the lowest dose of all the cell lines in our panel, did not manifest changes in global trimethylation of these histone marks under these conditions. Thus, effects of JIB-04 on global methylation levels appear to be dependent on both drug dose and cell line.

### JIB-04 dramatically alters the Ewing Sarcoma transcriptome

**Figure 3:**
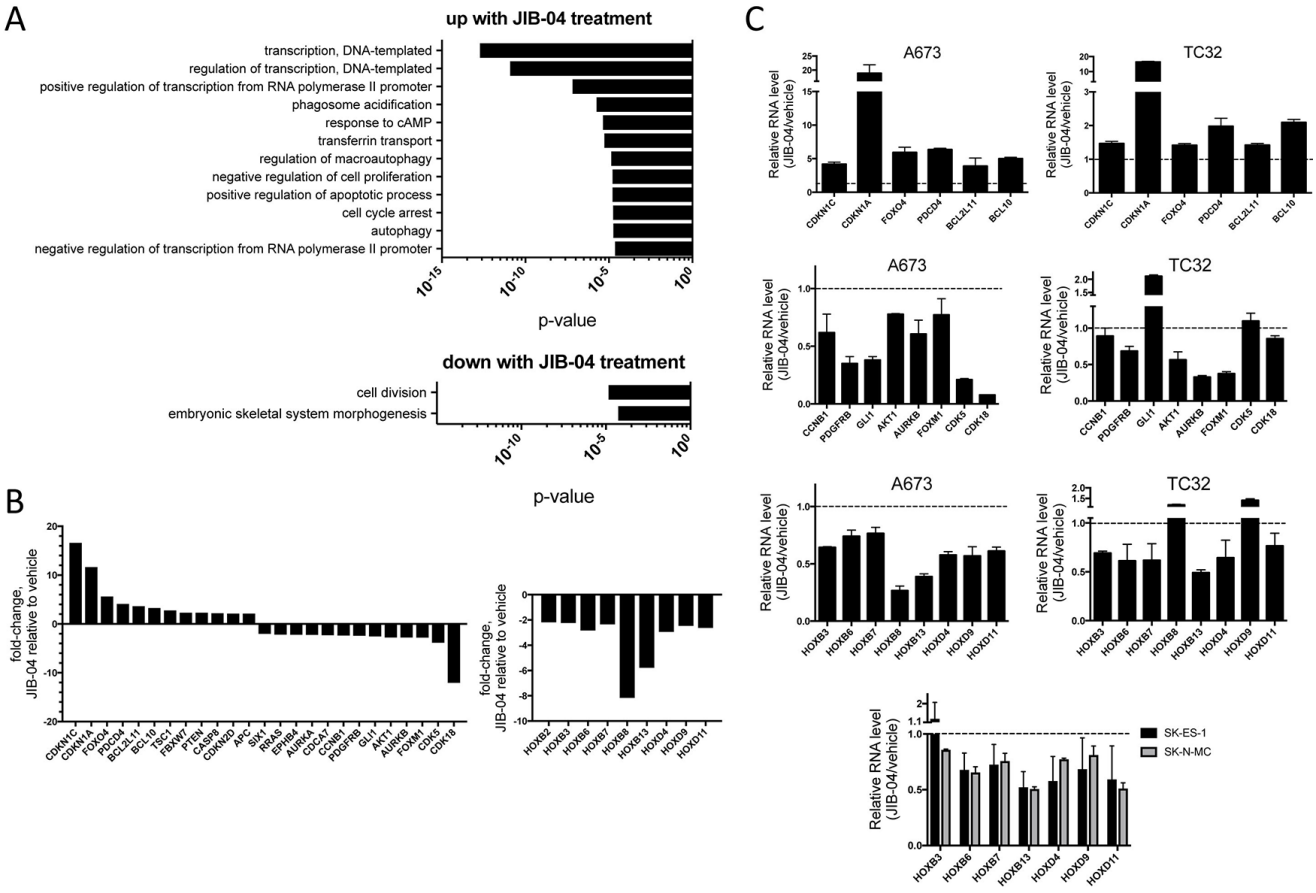
Effects of JIB-04 treatment on Ewing Sarcoma transcriptome **(A)** Gene Ontology (GO) terms significantly enriched (FDR<0.1) among genes upregulated (top panel) or downregulated (bottom panel) in expression 2-fold or more by treatment of A673 cells with JIB-04 (2 μM for 36 hours, relative to vehicle), as determined using DAVID. **(B)** Changes in RNA levels of candidate mediators of JIB-04 growth inhibitory effects, based on known gene function in cancer, and, for some genes, Ewing Sarcoma (see text for discussion; A673 RNA-seq data). Downregulation of HOXB and HOXD cluster genes by JIB-04 treatment (right panel, A673 RNA-seq data). Q-value <0.05 for all fold-changes shown, determined by ANOVA. **(C)** Quantification of selected upregulated and downregulated genes from A673 RNA-seq data in the indicated cell lines, using qRT-PCR (TC32: 0.3 μM JIB-04; SK-ES-1 and SK-N-MC: 1 μM; A673: 2 μM; RNA collected at 36 hours following treatment). Data are plotted as ratio of expression in JIB-04-treated cells to vehicle controls (mean and standard deviation from triplicate samples; dotted lines represent a ratio of 1 (no change in expression)).

**Figure 4:**
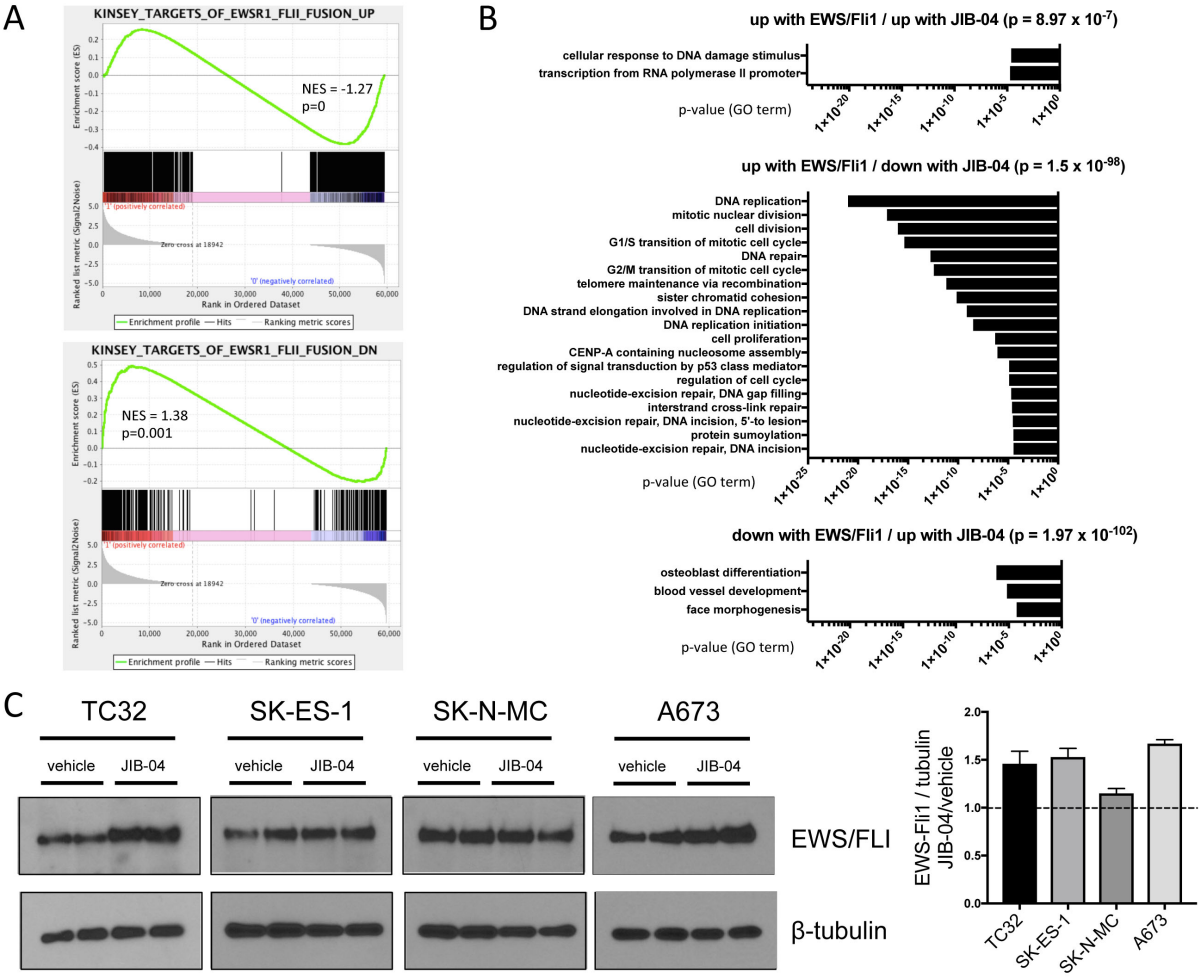
Effects of JIB-04 treatment on EWS/Fli1 expression signature and EWS/Fli1 protein levels **(A)** Gene Set Enrichment Analysis (GSEA) was performed with the JIB-04 transcriptome as the rank-ordered dataset (NES: normalized enrichment score). **(B)** The indicated significantly enriched gene subsets from GSEA analysis in “A” (p-values determined using hypergeometric test) were subjected to Gene Ontology (GO) analysis using DAVID; shown are GO terms enriched at FDR<0.1 (no significant pathway enrichment (FDR<0.1) was identified in the “down with EWS/Fli1/ down with JIB-04” subset). **(C)** EWS/Fli1 protein levels in vehicle and JIB-04-treated cells, determined 36 hours following treatment (TC32: 0.3 μM; SK-ES-1 and SK-N-MC: 1 μM; A673: 2 μM). Quantification of data (mean and SEM) from 2 independent experiments, each done in duplicate, is shown to the right (data plotted as EWS/Fli1 to tubulin densitometric ratios, normalized to vehicle-treated cells).

To get further insight into JIB-04 action in Ewing Sarcoma, we performed global transcriptome analysis on control (vehicle) and drug-treated A673 cells, using RNA-seq, under the same conditions as used for the above global histone mark analysis. This analysis revealed extensive alterations in gene expression upon drug treatment, including: 1657 genes significantly upregulated 2-fold or greater; and 1588 genes significantly downregulated 2-fold or greater (Q-value < 0.05; Supplementary Table 1). Gene Ontology (GO) analysis using DAVID (NIH Database for Annotation, Visualization, and Integrated Discovery) revealed, among gene groups most significantly upregulated upon JIB-04 treatment, at a false discovery rate (FDR) < 0.1, enrichment of anti-proliferative and pro-apoptotic pathways, consistent with JIB-04 growth inhibitory effects (Figure 3A). Additional biological processes significantly enriched among JIB-04 upregulated genes included processes related to transcription and gene regulation, as well as autophagy. Cellular factors involved in the control of gene expression represented a substantial proportion (roughly one third) of upregulated genes, and included many transcriptional regulators. GO biological processes significantly enriched among genes most significantly downregulated upon JIB-04 treatment, at a false discovery rate (FDR) < 0.1, included those related to proliferation, again consistent with the growth-inhibitory effects of JIB-04 (Figure 4A). Interestingly, also significantly downregulated were genes linked to developmental processes, a group that consisted largely of homeotic genes (see below).

More detailed examination of the transcriptomic changes revealed: upregulation of cell cycle inhibitors (CDKN1C, CDKN1A, CCNG2 and CDKN2D), pro-apoptotic factors (PDCD4, BCL2L11, BCL10 and CASP8) and tumor suppressors (FOXO4, TSC1, FBXW7, PTEN and APC); downregulation of cell cycle promoters (CDK5, CDK18, AURKA and AURKB) and oncogenes (FOXM1, AKT1, GLI1, PDGFRB, CCNB1, CDCA7, EPHB4, RRAS and SIX1) (Figure 3B, left panel). CDKN1C and a related member of the FOXO family, FOXO1, have previously been shown to inhibit Ewing Sarcoma growth [27, 28], while FOXM1, GLI1 and PDGFRB have been shown to be growth-promoting in Ewing Sarcoma [29–31]. Moreover, while not specifically functionally implicated in Ewing Sarcoma, PDCD4 has been identified as an EWS/Fli1-repressed gene, while AURKB, CCNB1, CDCA7 and AURKA have been identified as EWS/Fli1-induced genes [32], thus also suggesting potential roles in Ewing Sarcoma cell proliferation and survival. CDKN1A also appears to be negatively regulated by EWS/Fli1 [33].

A subset of genes from the above list (Figure 3B) was chosen for validation using qRT-PCR in A673 cells, and this analysis confirmed the changes seen by RNA-seq (Figure 3C). Interestingly, TC32 cells treated with JIB-04 at the lower drug dose that is growth-inhibitory in this cell line, also manifested altered expression of many of the same genes, although the changes tended to be lower in magnitude.

JIB-04 treatment also resulted in downregulation of a number of homeotic genes (Figure 3B). This group consisted largely of members of the HOXB and HOXD homeodomain clusters, which, interestingly, have recently been shown to be upregulated in Ewing Sarcoma [34]. Specifically, HOXB2, HOXB3, HOXB6, HOXB7, HOXB8, HOXB13, HOXD4, HOXD9 and HOXD11 were downregulated upon JIB-04 treatment in A673 cells (Figure 3B and 3C), and the majority of these were also downregulated in TC32, SK-ES-1 and SK-N-MC cells (Figure 3C). Notably, all but one of these (HOXD4) have previously been implicated in the promotion of cancer, and HOXD11 has specifically been demonstrated to be disease-promoting in Ewing Sarcoma [35, 36].

### JIB-04 interferes with the EWS/Fli1 gene expression program

To further examine JIB-04 transcriptomic effects more specifically on the EWS/Fli1-driven gene expression program in Ewing Sarcoma [32], we performed gene set enrichment analysis (GSEA). This revealed positive and negative regulation of subsets of both EWS/Fli1-induced and EWS/Fli1-repressed genes, with an overall trend toward repression of EWS/Fli1-induced genes, and induction of EWS/Fli1-repressed genes (Figure 4A). DAVID GO term analysis of these subsets revealed significant enrichment (FDR<0.1) of a number of biological processes (Figure 4B). Most strikingly, genes downregulated by JIB-04 treatment and also normally upregulated by EWS/Fli1 showed highly significant enrichment of processes related to cell proliferation (Figure 4B, middle panel). Genes in this subset included some of the same genes identified in our general transcriptomic analyses above (AURKA, AURKB, CCNB1 and FOXM1; Figure 3).

A number of different pharmacologic manipulations have been shown to alter EWS/Fli1 oncofusion levels in Ewing Sarcoma [37–39]. Since the oncofusion is known to promote cell proliferation and survival [40], downregulation of EWS/Fli1 levels could account for the observed opposing effects of JIB-04 on the EWS/Fli1-controlled transcriptome. We thus asked whether JIB-04 treatment affects EWS/Fli1 levels in Ewing Sarcoma cells. Interestingly, we found that JIB-04 treatment resulted in an approximately 1.5-fold increase in EWS/Fli1 protein levels in three of four cell lines (TC32, SK-ES-1 and A673; Figure 4C). Thus, JIB-04 globally interferes with the EWS/Fli1-regulated transcriptome, with an overall trend toward opposing effects, while at the same time slightly upregulating EWS/Fli1 levels.

### JIB-04 increases DNA damage

**Figure 5:**
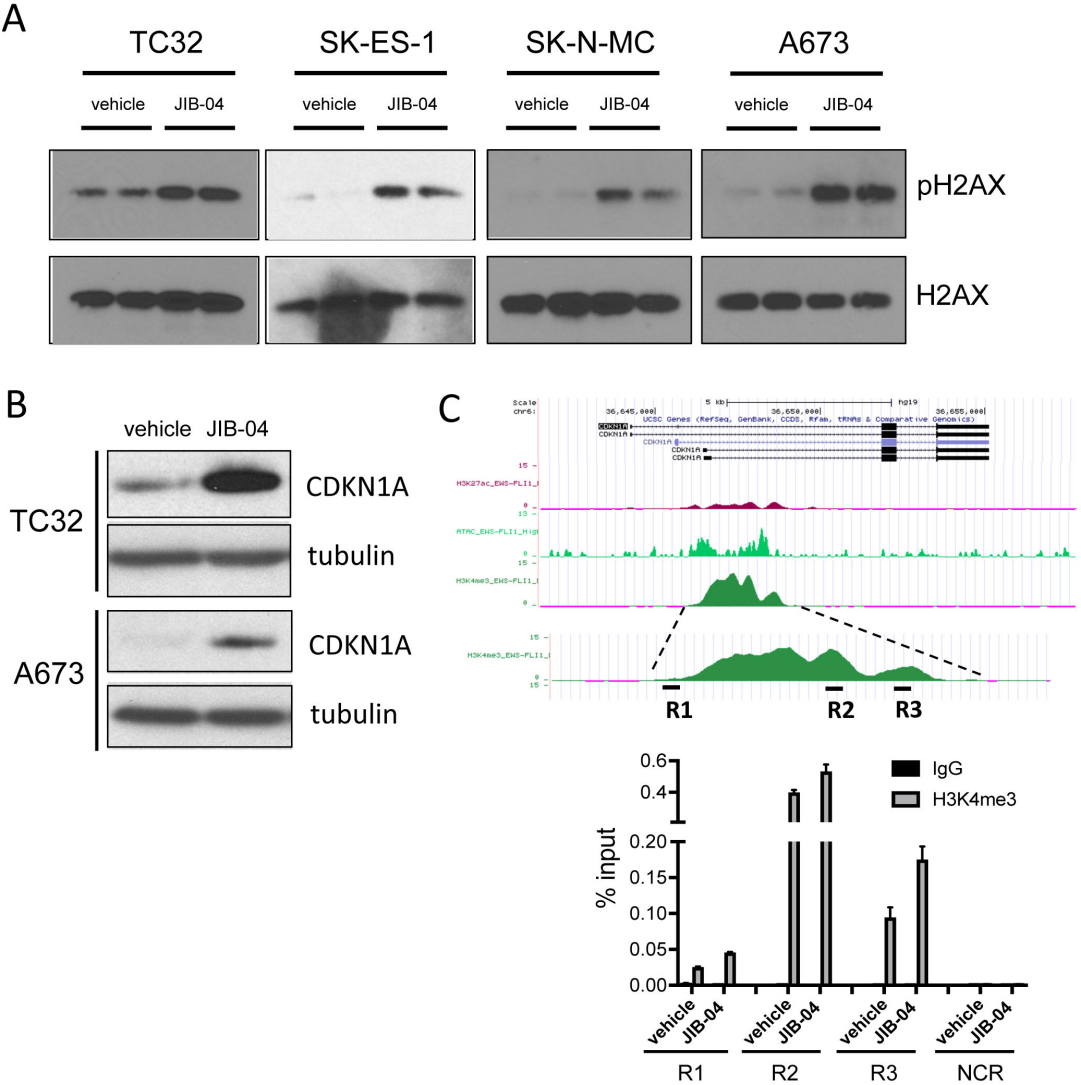
Effects of JIB-04 on DNA damage and CDKN1A expression **(A)** Levels of phosphorylated H2AX (pH2AX, Ser139) in vehicle and JIB-04-treated cells (TC32: 0.3 μM; SK-ES-1 and SK-N-MC: 1 μM; A673: 2 μM), as determined by immunoblotting of acid-extracted histones. **(B)** CDKN1A protein levels in TC32 cells (0.3 μM JIB-04 for 36 hours) and A673 (2 μM JIB-04 for 36 hours) cells, as determined by immunoblotting. **(C)** H3K4me3 ChIP-seq signal at CDKN1A promoter in A673 cells (data in UCSC browser from Tomazou et al [11]; top panel). Levels of H3K4me3 at the indicated CDKN1A promoter regions (R1-R3) in vehicle and JIB-04-treated cells (2 μM for 36 hours), as determined by ChIP-qPCR; data are plotted as mean and SEM of percent input from two independent ChIP experiments; NCR: negative control region (10 Kbp upstream of MCAM promoter [18]); see Supplementary Figure 2 for ChIP-qPCR signal in IgG samples as well as NCR.

**Figure 6:**
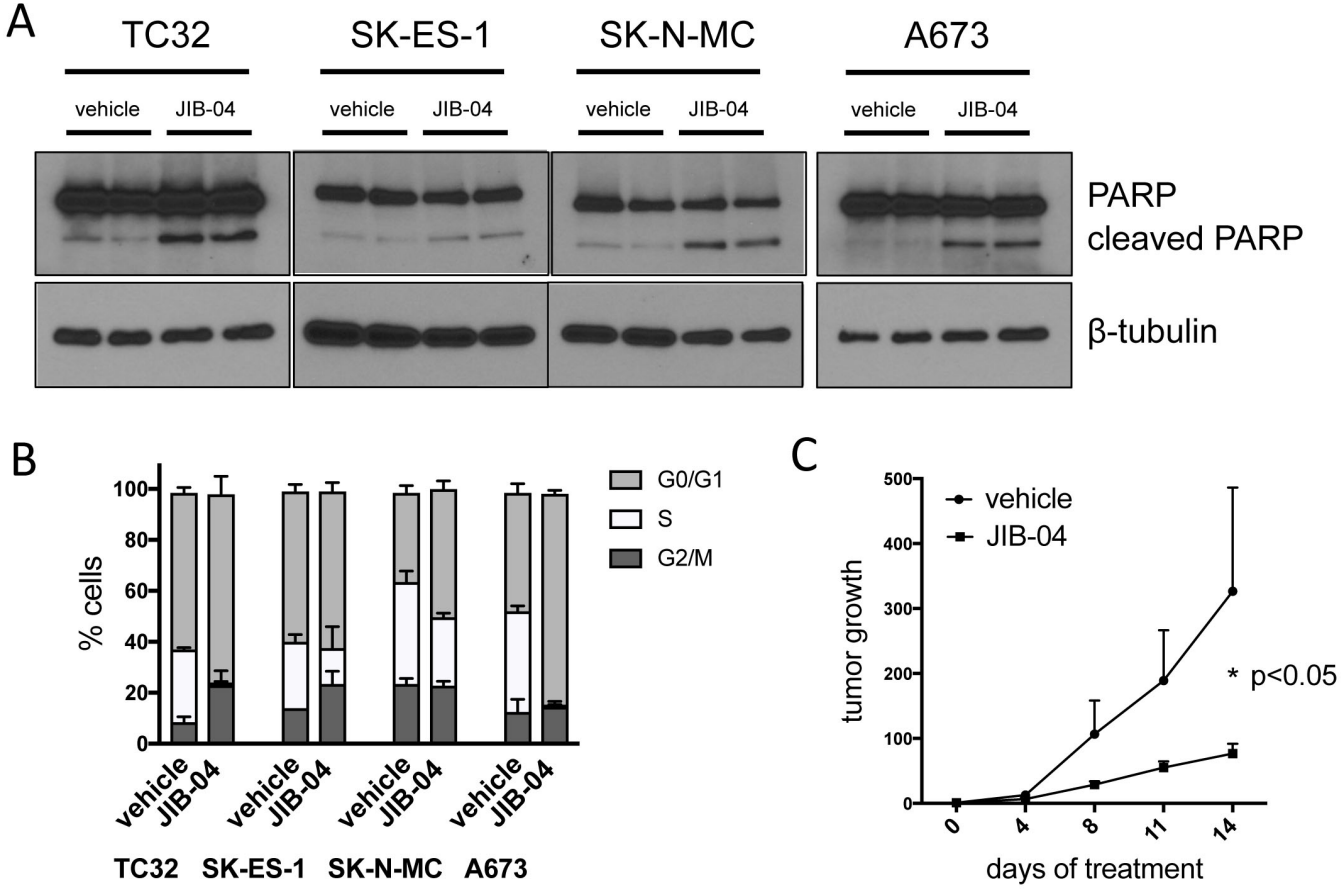
JIB-04 inhibits cell survival and cell cycle progression, and attenuates tumor growth *in vivo* **(A)** Apoptosis, as determined by PARP immunoblotting, in vehicle and JIB-04-treated cells (TC32: 0.3 μM; SK-ES-1 and SK-N-MC: 1 μM; A673: 2 μM; cells collected for analysis at 36 hours following treatment). **(B)** Cells were treated with vehicle or JIB-04 (TC32: 0.3 μM; SK-ES-1 and SK-N-MC: 1 μM; A673: 2 μM) for 36 hours, and cell cycle distribution was evaluated using EdU/PI staining and flow cytometry. Proportion of cells in G0/G1, S and G2/M is shown (mean and SEM of 2 independent experiments, each performed in duplicate). **(C)** Growth of control (vehicle; n=12) and drug-treated (JIB-04, 50 mg/Kg, daily, PO; n=13) Ewing Sarcoma xenografts arising from subcutaneously injected TC32 Ewing Sarcoma cells bearing a luciferase reporter, as quantified by intravital (IVIS) imaging. Data are shown as mean and standard error of signal (total flux) at given time point relative to signal at time of randomization (day 0; see Supplementary Figure 3A) for each tumor; p-value was calculated using two-way ANOVA with multiple comparisons and repeated measures. JIB-04-treated animals were well-appearing throughout the study, maintained body weight, and, similar to prior published studies with the drug [23], showed an increase in liver size (Supplementary Figure 3B).

Given the observed transcriptomic changes of upregulated DNA-damage response, but downregulated DNA-repair (Figure 4B), we examined the effects of JIB-04 upon DNA damage in Ewing Sarcoma cells, by quantifying levels of DNA damage-associated phospho-H2AX. This analysis revealed increased levels of DNA damage upon JIB-04 treatment in all cell lines examined (Figure 5A). DNA damage can be a potent inducer of CDKN1A expression, typically via p53 activation [41], and this could be a mechanism of CDKN1A induction in TC32 cells (Figure 3B and 6B), which contain wild-type p53. However, JIB-04 also leads to increased CDKN1A levels in p53-mutant A673 cells (Figures 3B and 6B), which, notably, do not induce CDKN1A in response to the DNA-damaging agent etoposide [42]. CDKN1A levels can additionally be subject to control by modulation of H3K4 methylation levels [43, 44]. To determine whether the latter mechanism could be contributing to induction of CDKN1A expression in A673 cells, we examined H3K4 methylation at the CDKN1A promoter by ChIP-PCR. This revealed increased H3K4me3 levels at three different promoter regions analyzed, but not a negative control region, upon JIB-04 treatment (Figure 5C). This suggests that inhibition of H3K4 promoter demethylation may be a contributing mechanism to CDKN1A induction by JIB-04. Thus, JIB-04 increases DNA damage, which in turn may be one, but not an exclusive, mechanism of CDKN1A induction in Ewing Sarcoma cells.

### JIB-04 inhibits cell cycle progression and cell survival, and attenuates tumor growth

The molecular and phenotypic alterations identified above, including deregulated expression of oncogenes and tumor suppressors, and compromised genome integrity, would be expected to impact cell cycle progression and cell survival, which in turn could be the basis for the observed growth inhibitory effects of JIB-04 (Figure 1). Indeed, we found that JIB-04 treatment results both in increased apoptosis, as indicated by increased PARP cleavage (Figure 6A), and impaired cell cycle progression, as indicated by diminution of the proportion of cells in S-phase (Figure 6B). Finally, in order to verify the inhibitory effects of JIB-04 in an *in vivo* context, we examined the effects of systemically delivered JIB-04 on tumor xenograft growth. TC32 Ewing Sarcoma cells, stably transduced with a luciferase reporter, were injected into the flanks of immunocompromised (NOD-SCID/Gamma) mice. Following randomization of animals into control and experimental groups (Supplementary Figure 3A), JIB-04 was administered daily via oral gavage at 50 mg/Kg. Compared to animals treated with vehicle alone, treatment with JIB-04 resulted in an approximately three-fold reduction of tumor growth (Figure 6C). Thus, systemically delivered JIB-04, via an oral route, is able to inhibit Ewing Sarcoma tumor growth in a xenograft model *in vivo*.

## DISCUSSION

Epigenetic alterations have recently emerged as playing very important roles in cancer [45]. Epigenetic mechanisms appear to play particularly prominent roles in the pathogenesis of pediatric cancers [6], which tend to have few genetic lesions [46]. In Ewing Sarcoma, a disease characterized by few genetic mutations [47], recent studies have shown that epigenetic mechanisms play a very important role in disease pathogenesis. Such mechanisms include provision of a unique cellular context for oncofusion action in the presumed cell of origin [8], as well as roles for the oncofusion itself in active remodeling of the epigenome to drive the sarcoma gene expression program [9, 10, 12]. Our own previous studies identified yet another level of epigenetic dysregulation in Ewing Sarcoma, involving the upregulation and action of a member of the Jumonji-domain histone demethylase (JHDM) family [16, 18]. In the present study, we examined the activity of a recently identified pan-JHDM pharmacologic inhibitor, JIB-04 [23], in Ewing Sarcoma. We find that JIB-04 inhibits the growth and viability of all Ewing Sarcoma cell lines tested under conditions of both low and high-density growth, while having little to no effect on mesenchymal stem cells, and that JIB-04, administered orally, inhibits the growth of Ewing Sarcoma tumor xenografts. These studies provide evidence in support of JIB-04 efficacy against Ewing Sarcoma, and suggest that this compound warrants further investigation in this disease.

In keeping with its pan-JHDM inhibitory activity and the epigenetic dependency of EWS/Fli1-driven Ewing Sarcoma pathogenesis, JIB-04 exerts profound and diverse effects on Ewing Sarcoma cells. JIB-04 has the potential to inhibit all members of the JHDM family, which include factors modulating the activity of multiple activating and repressive histone methyl marks critical to gene expression, cell proliferation and maintenance of genomic integrity. Since its discovery and initial characterization [23], JIB-04 has been used as an inhibitor of the H3K9/K36 demethylase activity of KDM4A in leukemia [48], the H3K9 demethylase activity of KDM3B in lung cancer [49], and the H3K4 demethylase activity of members of the KDM5 family in breast cancer and glioblastoma [50, 51]. In Ewing Sarcoma, we find evidence of activity against multiple histone methyl marks controlled by different JHDM families (eg: H3K27 demethylation/ KDM6 family, H3K4 demethylation/ KDM5 family). It is likely that the molecular alterations, including changes in global levels of histone methyl marks and transcriptome changes, and phenotypic effects, including changes in cell cycle progression, apoptosis and levels of DNA damage, observed in our studies represent a sum total of impairment of JHDM activity in multiple biological processes.

Cellular levels of H3K4 and H3K27 trimethylation can increase as a consequence of DNA damage [52, 53]. However, TC32 cells accumulate DNA damage of similar magnitude to the other cell lines, but do not manifest increased total H3K4me3 or/and H3K27me3. This suggests that increased DNA damage is not likely to be the sole mechanism for the increased histone trimethylation observed in our studies. Rather, increased H3K4/27 trimethylation probably reflects JHDM inhibition by the drug at the higher doses required to achieve effects on cell growth and viability in SK-ES-1, SK-N-MC and A673 cells. Altered H3K4/27 trimethylation on the other hand is likely to be the basis of at least some of the observed transcriptome changes. For example, increased H3K27 trimethylation provides a plausible mechanism for the observed downregulation of HOXD gene expression, as the HOXD locus is normally essentially devoid of this repressive mark in Ewing Sarcoma cells [34].

TC32 cells respond to JIB-04 differently than the other cell lines studied. They are growth/viability-inhibited at lower doses, and, at such doses, do not manifest detectable changes in total H3K4/9/27 trimethylation. Notably, TC32 cells are the only cell line in our panel with an intact p53 response. One possible explanation for the findings is that, in the context of an intact p53 pathway, the DNA damage sustained in TC32 cells at low drug doses is sufficient to trigger growth arrest and apoptosis. In the other cell lines with non-functional p53, this may not be sufficient to compromise cell growth/viability, and additional effects of the drug, achieved at higher doses and reflected in increased histone trimethylation, may be necessary. The increased DNA damage observed in all cell lines studied could be due to inhibition of JHDM functions in DNA damage/repair (eg: KDM5A [54]), transcriptional stress due to elevated EWS/Fli1 levels (see discussion below), or other effects of JIB-04 unknown at this time. The consistently increased DNA damage observed upon JIB-04 treatment suggests that the drug could have favorable combinatorial activity with other DNA damaging agents (eg: temozolomide) or/and inhibitors of DNA repair (eg: PARP inhibitors), the former observed in glioblastoma [50].

Our studies indicate that JIB-04 exerts profound effects on the EWS/Fli1-controlled transcriptome. The effects resemble, but are not as dramatic as, those enacted by pharmacologic inhibition of LSD1 (a non-Jumonji histone demethylase with dual specificity for H3K4me2/3 and H3K9me1/2) in Ewing Sarcoma, which showed near-complete opposition of both EWS/Fli1-upregulated and downregulated transcriptomes [15]. Using similar GSEA analysis, the effects of JIB-04 on the EWS/Fli1 up/down transcriptomes are more mixed. However, in some similarity to effects of LSD1 inhibition, there is an overall trend toward greater opposition of EWS/Fli1-driven gene expression by JIB-04, particularly in the case of pro-proliferative genes. Since this is not associated with downregulation of EWS/Fli1 protein levels in the cells, these findings suggest that JIB-04 could be acting in part by uncoupling EWS/Fli1 from its transcriptome. While our studies cannot make conclusions regarding direct versus indirect effects of JIB-04 on expression of individual genes, these observations further suggest that JHDMs could be contributing specifically and importantly to EWS/Fli1-driven gene expression.

Of interest is the observation that JIB-04 treatment leads to modestly increased EWS/Fli1 protein levels in most cell lines studied. This is particularly intriguing in the context of upregulation of transcription-associated processes and DNA damage, and downregulation of DNA repair, as indicated by our transcriptome analyses. EWS/Fli1 is a known inducer of DNA damage [55], and Ewing Sarcoma cells are relatively deficient in DNA repair [56]. Moreover, recent studies indicate that EWS/Fli1 imposes transcriptional stress on the cell [57]. Together, these findings suggest that another mechanism by which JIB-04 could be acting in Ewing Sarcoma is exacerbation of EWS/Fli1 genotoxic effects. This could be an important mechanism of growth/survival impairment in TC32 cells, which show increased levels of EWS/Fli1 and DNA damage, but not H3K4/9/27 trimethylation.

In summary, our studies demonstrate activity of the pan-JHDM inhibitor JIB-04 in Ewing Sarcoma, and indicate the existence of multiple mechanisms contributing to its effects. The studies further suggest the existence of mechanistic intersections between the biology of JHDMs and the action of the EWS/Fli1 oncofusion, reinforcing the importance of epigenetic mechanisms in the pathogenesis of this disease.

## MATERIALS AND METHODS

### Cell lines and drugs

The Ewing Sarcoma cell lines and human Mesenchymal Stem Cells (hMSC; Lonza) used in this study have been previously described [17, 18, 24]. All Ewing Sarcoma cell lines were authenticated at our institution by STR profiling, and all cell lines were repeatedly verified to be mycoplasma-free. JIB-04 was obtained from ApexBio (active E isomer; compound activity was verified by comparing growth inhibition to JIB-04 samples kindly provided by the Martinez laboratory at the University of Texas Southwestern Medical Center [23] (Supplementary Figure 1)). For *in vitro* studies, JIB-04 was dissolved in DMSO; for *in vivo* (animal tumor) studies, JIB-04 was administered by oral gavage as an aqueous suspension in 12.5% DMSO and 12.5% Cremophor EL [23].

### *In vitro* assays of drug sensitivity

Response of Ewing Sarcoma cells to JIB-04 treatment under high density growth conditions was evaluated using an MTT assay, as previously described [24]. IC50 values were determined via a non-linear regression plot performed using the GraphPad statistical software package. Response of Ewing Sarcoma cells to JIB-04 under low density growth conditions was evaluated using a clonogenic assay, also as described [24]. Briefly, 500 cells per well were plated in triplicate in 6-well plates, and drug or vehicle control was administered as described in Figure 1 legend. Colonies were visualized using crystal violet staining.

### Cell cycle analysis

Following treatment with vehicle or JIB-04 as indicated, cells were incubated in 10 μM EdU for 60 minutes, followed by trypsinization, harvest, and processing using the Click-iT Plus EdU Flow Cytometry Assay Kit (Life Technologies, C10634, Alexa Fluor 647), per manufacturer instructions. This was followed by treatment with RNAse (25 μg/ml) and propidium iodide (25 μg/ml), and analysis on the Gallios flow cytometer (Beckman Coulter).

### Protein expression and histone mark analysis

Protein expression levels were determined as previously described [17]. Primary antibodies used were: Fli1 (BD Biosciences, #554266, 1:250); PARP (Cell Signaling Technology, #9542, 1:1000); CDKN1A (Cell Signaling Technology, #2946, 1:1000); and α-tubulin (Sigma, #T5168, 1:20,000). Global histone mark analysis was performed also as previously described [16]. Primary antibodies used were: H3K4me3 (Cell Signaling Technologies, #9751, 1:1000); H3K9me3 (Cell Signaling Technologies, #13969, 1:1000); H3K27me3 (Cell Signaling Technologies, #9733, 1:1000); phospho-H2AX (Ser139; Cell Signaling Technologies, #9718, 1:1000); H2AX (Cell Signaling Technologies, #2595S, 1:1000); and H3 (Abcam, #1791, 1:1000).

### Gene expression analysis and validation

A673 cells were treated with vehicle (DMSO) or 2 μM JIB-04 for 36 hours, each group in quadruplicate. RNA was isolated using TRIzol extraction, and further purified using the Qiagen MinElute column kit. Samples were submitted to the University of Colorado Cancer Center Microarray and Genomics shared resource for analysis of RNA quality, library preparation, and directional mRNA next-generation sequencing at 50 cycles of single-end reads on an Illumina Hi-Seq 4000 instrument. Sequencing data were processed through a custom computational pipeline consisting of the open-source gSNAP, Cufflinks, and R for alignment and discovery of differential gene expression [58, 59]. Fragments per kilobase of exon per million mapped reads (FPKM) were used for comparison of transcript levels, and significant differences in gene expression were calculated using ANOVA in R. Functional annotation analysis was performed using the National Institutes of Health Database for Annotation, Visualization, and Integrated Discovery (DAVID) public on-line tool (http://david.abcc.ncifcrf.gov/) using Biological Process Gene Ontology (GO) terms. Gene Set Enrichment Analysis was performed using GSEA software (PMID: 16199517) and MSigDB C2 gene sets and published signatures. Gene sets with p < 0.05 (after 1000 gene set permutations) were deemed to be enriched in each group. Deposition of the expression profiling data into the NCBI Gene Expression Omnibus database has been initiated (accession number pending). Expression of selected genes was also analyzed in A673, TC32, SK-ES-1 and SK-N-MC cells using qRT-PCR, performed as previously described [18]; primers used are listed in Supplementary Materials (Supplementary Table 2).

### Chromatin immunoprecipitation

A673 cells were treated with vehicle (DMSO) or 2 μM JIB-04 for 36 hours. Chromatin immunoprecipitation analysis followed by qPCR (ChIP-qPCR), to compare H3K4me3 levels at the CDKN1A promoter in vehicle and drug-treated cells was performed essentially as previously described [18], with the following modifications: 4 x 10^6^ cells were resuspended in 500ul of lysis buffer, and sonicated in the Diagenode Bioruptor for 25 cycles (30sec on/90sec off on High setting); lysate was diluted up to 4x the volume before clearing with Protein A/G beads; 5 μg of control (rabbit IgG; Santa Cruz, sc-2027) and specific (H3K4me3; Invitrogen, #49-1005) antibodies were used for immunoprecipitation. Primers used for qPCR are listed in Supplementary Materials (Supplementary Table 2).

### Animal tumor xenograft studies

Tumor studies were performed according to an institutionally approved animal protocol. Ewing Sarcoma TC32 cells were labeled for intravital (IVIS) imaging via transduction with a lentiviral GFP/luciferase dual reporter (SFG-NES-TGL; [60]) and subsequent sorting for GFP expression using flow cytometry. 1 x 10^6^ GFP/luciferase-tagged TC32 cells, mixed 1:1 with Matrigel, were then injected subcutaneously into the flank of immunocompromised (NOD-SCID/Gamma) mice. Tumor growth was monitored using IVIS imaging following administration of luciferin. On day 4 following injection, animals were randomized into control (vehicle) and treatment (JIB-04) groups based on IVIS imaging/quantification. Drug treatment was initiated on day 5, and continued until cessation of the experiment. All animal studies were approved by our Institutional Animal Care and Use Committee.

## SUPPLEMENTARY MATERIALS FIGURES AND TABLES






