# Supplementary material for: The Jumonji-domain histone demethylase inhibitor JIB-04 deregulates oncogenic programs and increases DNA damage in Ewing Sarcoma, resulting in impaired cell proliferation and survival, and reduced tumor growth
Source: Oncotarget. 2018 Sep 4;9(69):33110–23. doi: 10.18632/oncotarget.26011 (PMC6145692; doi:10.18632/oncotarget.26011)
Supplement: Supplementary file 1 [file oncotarget-09-33110-s001.pdf]

The Jumonji-domain histone demethylase inhibitor JIB-04 deregulates oncogenic programs and increases DNA damage in Ewing Sarcoma, resulting in impaired cell proliferation and survival, and reduced tumor growth

SUPPLEMENTARY MATERIALS

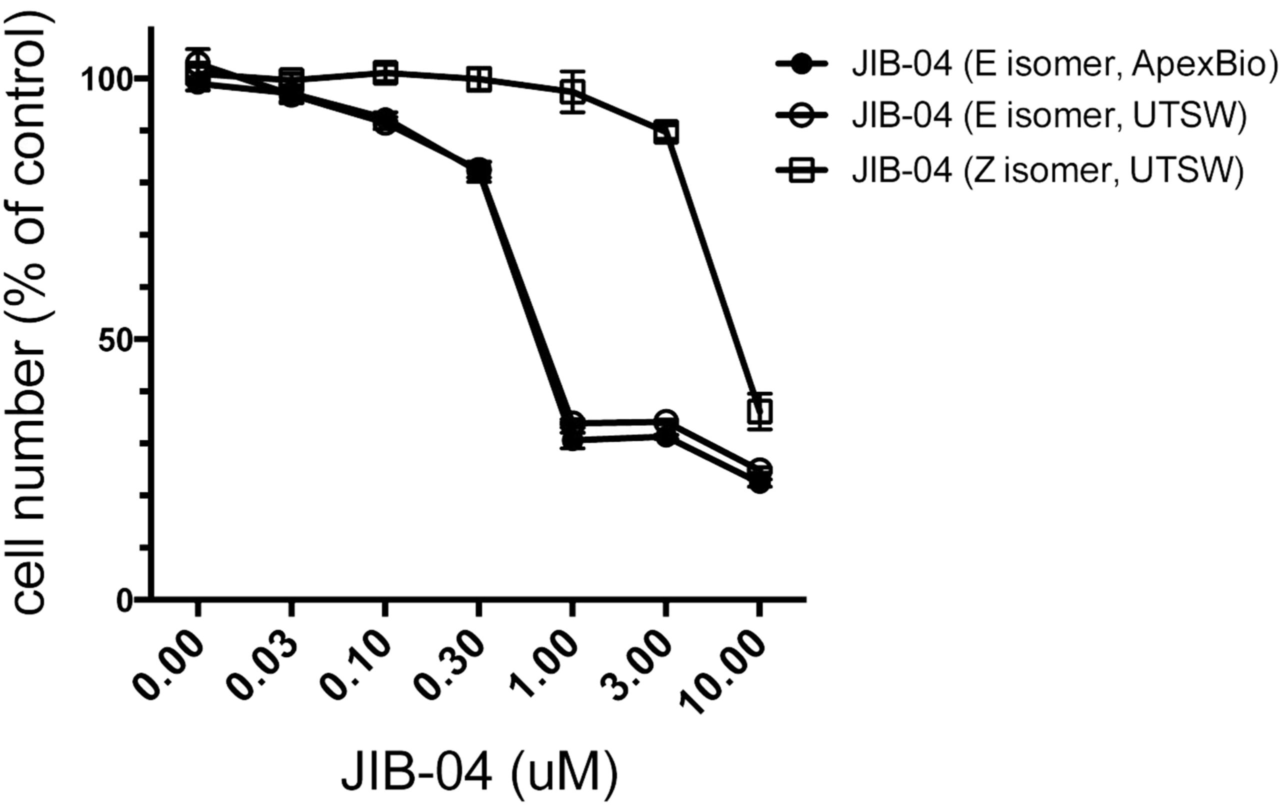

Supplementary Figure 1: One day following plating, A673 cells were treated for 48 hours with different concentrations of the indicated compound (JIB-04 purchased from ApexBio or JIB-04 (active (E) or inactive (Z) isomer) kindly provided by the Martinez laboratory at UTSW. Cell numbers at the end of the experiment were quantified using an MTT assay, and were normalized to vehicle-treated cells. Results represent the mean and standard deviation (SD) of triplicate platings for each condition.

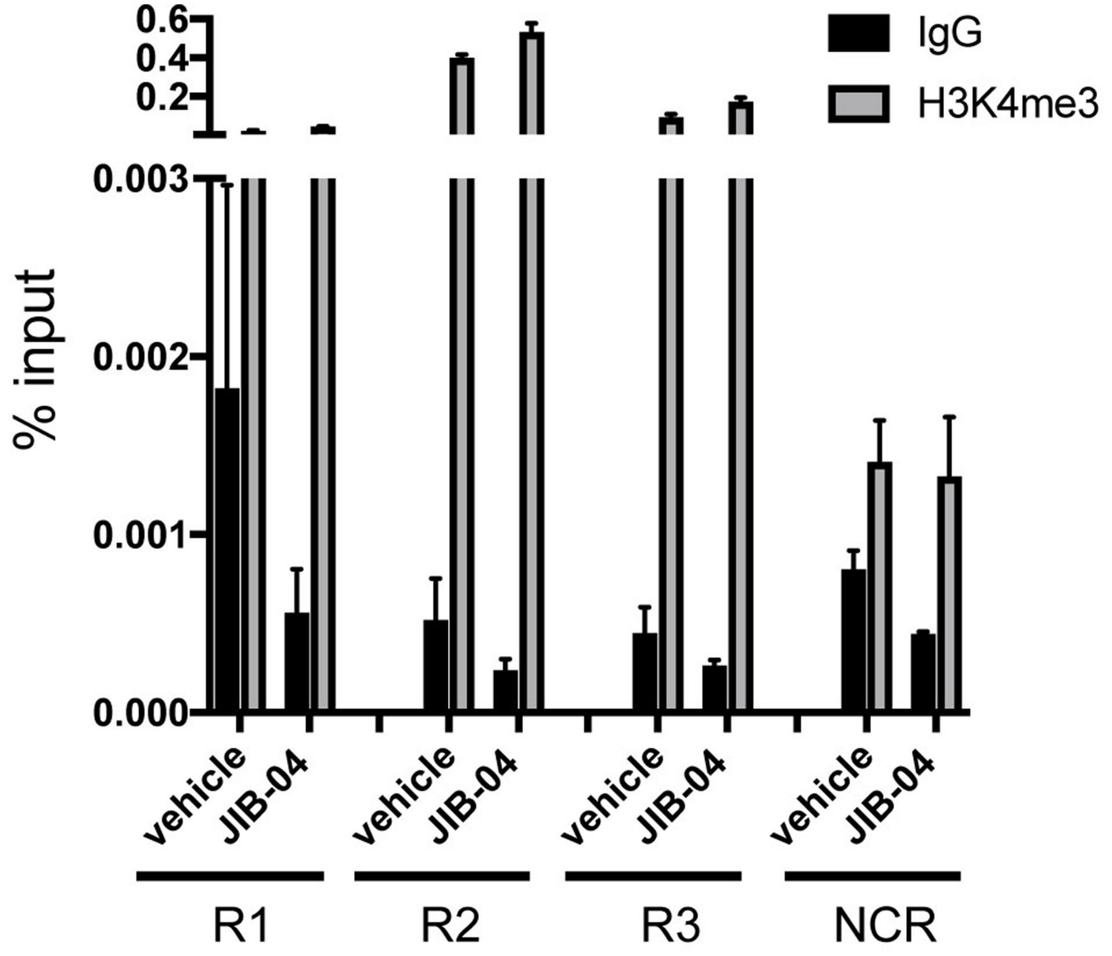

Supplementary Figure 2: Same data as in Figure 5C, with y-axis adjusted to visualize ChIP-qPCR signal in IgG controls and negative control region (NCR).

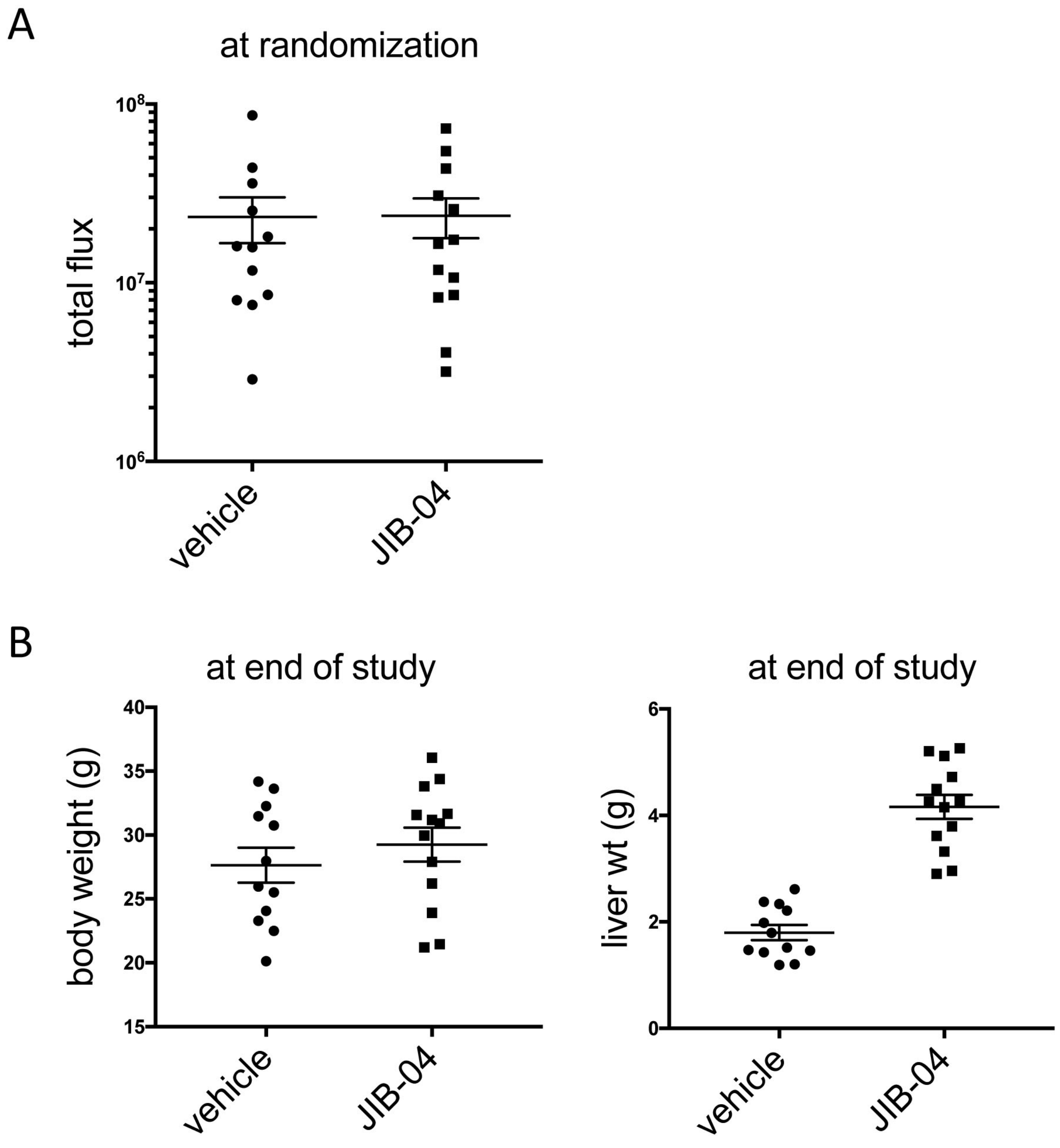

Supplementary Figure 3: (A) Total flux at randomization in control (vehicle; n=12) and drug-treated (JIB-04; n=13) groups, for data shown in Figure 6C. Individual values, mean, and standard error of the mean for each group are shown. **(B)** Body and liver weights of animals at end of study (mean and standard error of the mean; g: grams).

Supplementary Table 1: Transcripts changed 2-fold or more upon JIB-04 treatment in A673 cells (Q-value < 0.05)

See Supplementary File 1

Supplementary Table 2: Primers used

See Supplementary File 2
